# Structure of a putative NTP pyrophosphohydrolase: YP_001813558.1 from *Exiguobacterium sibiricum* 255-15

**DOI:** 10.1107/S1744309110025534

**Published:** 2010-08-04

**Authors:** Gye Won Han, Marc-André Elsliger, Todd O. Yeates, Qingping Xu, Alexey G. Murzin, S. Sri Krishna, Lukasz Jaroszewski, Polat Abdubek, Tamara Astakhova, Herbert L. Axelrod, Dennis Carlton, Connie Chen, Hsiu-Ju Chiu, Thomas Clayton, Debanu Das, Marc C. Deller, Lian Duan, Dustin Ernst, Julie Feuerhelm, Joanna C. Grant, Anna Grzechnik, Kevin K. Jin, Hope A. Johnson, Heath E. Klock, Mark W. Knuth, Piotr Kozbial, Abhinav Kumar, Winnie W. Lam, David Marciano, Daniel McMullan, Mitchell D. Miller, Andrew T. Morse, Edward Nigoghossian, Linda Okach, Ron Reyes, Christopher L. Rife, Natasha Sefcovic, Henry J. Tien, Christine B. Trame, Henry van den Bedem, Dana Weekes, Keith O. Hodgson, John Wooley, Ashley M. Deacon, Adam Godzik, Scott A. Lesley, Ian A. Wilson

**Affiliations:** a Joint Center for Structural Genomics, http://www.jcsg.org, USA; b Department of Molecular Biology, The Scripps Research Institute, La Jolla, CA, USA; c Department of Chemistry and Biochemistry, University of California Los Angeles, Los Angeles, CA, USA; d Stanford Synchrotron Radiation Lightsource, SLAC National Accelerator Laboratory, Menlo Park, CA, USA; e MRC Laboratory of Molecular Biology, Hills Road, Cambridge, England; f Center for Research in Biological Systems, University of California, San Diego, La Jolla, CA, USA; g Program on Bioinformatics and Systems Biology, Sanford–Burnham Medical Research Institute, La Jolla, CA, USA; h Protein Sciences Department, Genomics Institute of the Novartis Research Foundation, San Diego, CA, USA; i Photon Science, SLAC National Accelerator Laboratory, Menlo Park, CA, USA

**Keywords:** structural genomics, putative NTP pyrophosphohydrolase, MazG nucleotide pyrophosphohydrolase, dUTPases

## Abstract

The crystal structure of a putative NTPase, YP_001813558.1 from *Exiguo­bacterium sibiricum* 255-15 (PF09934, DUF2166) was determined to 1.78 Å resolution. YP_001813558.1 and its homologs (dimeric dUTPases, MazG proteins and *HisE*-encoded phosphoribosyl ATP pyrophosphohydrolases) form a superfamily of all-α-helical NTP pyrophosphatases. In dimeric dUTPase-like proteins, a central four-helix bundle forms the active site. However, in YP_001813558.1, an unexpected intertwined swapping of two of the helices that compose the conserved helix bundle results in a ‘linked dimer’ that has not previously been observed for this family. Interestingly, despite this novel mode of dimerization, the metal-binding site for divalent cations, such as magnesium, that are essential for NTPase activity is still conserved. Furthermore, the active-site residues that are involved in sugar binding of the NTPs are also conserved when compared with other α-helical NTPases, but those that recognize the nucleotide bases are not conserved, suggesting a different substrate specificity.

## Abstract 2

The crystal structure of a putative NTP pyrophosphohydrolase, YP_001813558.1 from *E. sibiricum*, reveals a novel segment-swapped linked-dimer assembly.

## 1. Introduction

Nucleoside triphosphate pyrophosphatases (or pyrophospho­hydrolases; NTPases) perform the important function of hydrolyzing the α–β phosphodiester bond of nucleoside triphosphates (NTPs) and are often involved in removing noncanonical nucleotide triphos­phates to prevent their incorporation into DNA or RNA (Bessman *et al.*, 1996 ▶; Wu *et al.*, 2007 ▶; Hwang *et al.*, 1999 ▶; Minasov *et al.*, 2000 ▶). dUTP pyrophosphatase (dUTPase; EC 3.6.1.23) catalyzes the hydrolysis of dUTP to dUMP and pyrophosphate. The available dUTPase structures are classified into three distinct groups based on their oligomeric state: trimeric, dimeric and monomeric. The crystal structures of trimeric dUTPases from *Escherichia coli* (Cedergren-Zeppezauer *et al.*, 1992 ▶; Larsson *et al.*, 1996 ▶), human (Mol *et al.*, 1996 ▶) and two mammalian retroviruses (Prasad *et al.*, 1996 ▶; Dauter *et al.*, 1999 ▶) possess an all-β fold. Monomeric dUTPases contain all five of the characteristic sequence motifs present in trimeric dUTPases, but they are arranged in a different order. The monomeric enzyme from Epstein–Barr virus (EVB; Tarbouriech *et al.*, 2005 ▶) also adopts an all-β fold and contains three domains and an active site that is very similar to those of trimeric dUTPases. Dimeric dUTPases, such as those from *Trypanosoma cruzi* (Harkiolaki *et al.*, 2004 ▶) and *Campylobacter jejuni* (Moroz *et al.*, 2004 ▶), differ from the monomeric and trimeric forms and adopt an all-α topology, indicating a different evolutionary origin.

Dimeric dUTPase and MazG proteins are members of the all-α-helical NTP pyrophosphatase SCOP superfamily (Murzin *et al.*, 1995 ▶; Andreeva *et al.*, 2008 ▶), which also contains the *HisE*-encoded phosphoribosyl ATP pyrophosphohydolase (PRATP-PH) family (Moroz *et al.*, 2005 ▶; Javid-Majd *et al.*, 2008 ▶). The α-helical NTP pyrophos­phatases share a highly conserved four-helix bundle, one face of which forms the active site, while the other participates in oligomer assembly (Harkiolaki *et al.*, 2004 ▶; Moroz *et al.*, 2004 ▶). In some cases, the four-helix bundle forms upon dimerization (Harkiolaki *et al.*, 2004 ▶) while, in others, it is contained within a single protomer (Moroz *et al.*, 2004 ▶).

Here, we report the crystal structure of NTPase YP_001813558.1 from the extremophile *Exiguobacterium sibiricum* 255-15 (PF09934, DUF2166), which was originally isolated from the Siberian permafrost (Vishnivetskaya *et al.*, 2000 ▶). The structure reveals an interesting variant of the all-α-helical NTP pyrophosphatase fold family that contains an unusual intertwined swapping of helical segments, resulting in an obligatory dimer that cannot dissociate without unfolding of the monomers. This novel ‘linked dimer’ defines a new subfamily of the α-helical NTP pyrophosphatase fold and is distinct from other previously observed domain-swapped dimers. The YP_001813558.1 gene of *E. sibiricum* 255-15 encodes a protein with a molecular weight of 19.1 kDa (residues 2–170) and a calculated isoelectric point of 4.93. The structure was determined using the semiautomated high-throughput pipeline of the Joint Center for Structural Genomics (JCSG; Lesley *et al.*, 2002 ▶) as part of the NIGMS Protein Structure Initiative (PSI).

## 2. Materials and methods

### 2.1. Protein production and crystallization

Clones were generated using the Polymerase Incomplete Primer Extension (PIPE) cloning method (Klock *et al.*, 2008 ▶). The gene encoding YP_001813558.1 (gi|172057098; UniProt B1YMF4) was amplified by polymerase chain reaction (PCR) from *E. sibiricum* 255-­15 genomic DNA using *PfuTurbo* DNA polymerase (Stratagene) and I-PIPE (Insert) primers (forward primer, 5′-ctgtacttccagggcATGAAACAACCGAACTACTATCAGGACG-3′; reverse primer, 5′-aattaagtcgcgttaTGCTTTTTCTTTCATTTGGCGCACTAC-3′; target sequence in upper case) that included sequences for the predicted 5′ and 3′ ends. The expression vector pSpeedET, which encodes an amino-terminal tobacco etch virus (TEV) protease-cleavable expression and purification tag (MGSDKIHHHHHHENLYFQ/G), was PCR-amplified with V-PIPE (Vector) primers (forward primer, 5′-taacgcgacttaattaactcgtttaaacggtctccagc-3′; reverse primer, 5′-gcc­ctggaagtacaggttttcgtgatgatgatgatgatg-3′). The V-PIPE and I-PIPE PCR products were mixed to anneal the amplified DNA fragments together. *Escherichia coli* GeneHogs (Invitrogen) competent cells were transformed with the V-PIPE/I-PIPE mixture and dispensed onto selective LB–agar plates. The cloning junctions were confirmed by DNA sequencing. Expression was performed in a selenomethionine-containing medium with suppression of normal methionine synthesis (Van Duyne *et al.*, 1993 ▶). At the end of fermentation, lysozyme was added to the culture to a final concentration of 250 µg ml^−1^ and the cells were harvested and frozen. After one freeze–thaw cycle, the cells were homogenized in lysis buffer [50 m*M* HEPES pH 8.0, 50 m*M* NaCl, 10 m*M* imidazole, 1 m*M* tris(2-carboxyethyl)phos­phine–HCl (TCEP)] and the lysate was clarified by centrifugation at 32 500*g* for 30 min. The soluble fraction was passed over nickel-chelating resin (GE Healthcare) pre-equilibrated with lysis buffer, the resin was washed with wash buffer [50 m*M* HEPES pH 8.0, 300 m*M* NaCl, 40 m*M* imidazole, 10%(*v*/*v*) glycerol, 1 m*M* TCEP] and the protein was eluted with elution buffer [20 m*M* HEPES pH 8.0, 300 m*M* imidazole, 10%(*v*/*v*) glycerol, 1 m*M* TCEP]. The eluate was buffer-exchanged with TEV buffer (20 m*M* HEPES pH 8.0, 200 m*M* NaCl, 40 m*M* imidazole, 1 m*M* TCEP) using a PD-10 column (GE Healthcare) and incubated with 1 mg TEV protease per 15 mg eluted protein for 2 h at 295 K and 18 h at 277 K. The protease-treated eluate was run over nickel-chelating resin (GE Healthcare) pre-equilibrated with HEPES crystallization buffer (20 m*M* HEPES pH 8.0, 200 m*M* NaCl, 40 m*M* imidazole, 1 m*M* TCEP) and the resin was washed with the same buffer. The flowthrough and wash fractions were combined and concentrated to 16.5 mg ml^−1^ by centrifugal ultrafiltration (Millipore) for crystallization trials. YP_001813558.1 was crystallized using the nanodroplet vapor-diffusion method (Santarsiero *et al.*, 2002 ▶) with standard JCSG crystallization protocols (Lesley *et al.*, 2002 ▶). Sitting drops composed of 200 nl protein mixed with 200 nl crystallization solution were equilibrated against a 50 µl reservoir at 277 K for 29 d prior to harvest. The crystallization reagent that produced the YP_001813558.1 crystal used for structure solution consisting of 1.4 *M* trisodium citrate and 0.1 *M* HEPES pH 7.5. For crystal diffraction screening and data collection, 1,2-ethanediol (ethylene glycol) was diluted to 20%(*v*/*v*) using reservoir solution and then added to the crystal drop in a 1:1 ratio as a cryoprotectant. Initial screening for diffraction was carried out using the Stanford Automated Mounting (SAM; Cohen *et al.*, 2002 ▶) system and an X-ray microsource (Miller & Deacon, 2007 ▶) installed at the Stanford Synchrotron Radiation Lightsource (SSRL, Menlo Park, California, USA). The data were indexed in the monoclinic space group *C*2. The oligomeric state of YP_001813558.1 was determined using a 1 × 30 cm Superdex 200 column (GE Healthcare) coupled with miniDAWN static light-scattering (SEC/SLS) and Optilab differential refractive-index detectors (Wyatt Technology). The mobile phase consisted of 20 m*M* Tris pH 8.0, 150 m*M* NaCl and 0.02%(*w*/*v*) sodium azide. The molecular weight was calculated using *ASTRA* v.5.1.5 software (Wyatt Technology).

### 2.2. Data collection, structure solution and refinement

Multiple-wavelength anomalous diffraction (MAD) data at wavelengths corresponding to the low-energy remote (λ_1_) and inflection point (λ_2_) of a selenium MAD experiment were collected on beamline 8.2.2 at Advanced Light Source (ALS, Berkeley, California, USA). The data were collected at 100 K using an ADSC Q315 CCD detector. Collection of the two wavelengths was interleaved using a 10° wedge size. The MAD data were integrated and reduced using *MOSFLM* (Leslie, 1992 ▶) and scaled with the program *SCALA* (Collaborative Computational Project, Number 4, 1994 ▶). The diffraction data were anisotropic, with a faster falloff along a*. The selenium substructure solution, phasing and density modification were performed with *SHELXD* (Sheldrick, 2008 ▶) and *autoSHARP* (Vonrhein *et al.*, 2007 ▶), resulting in a mean figure of merit of 0.30 with four selenium sites. Automatic model building was performed with *ARP*/*wARP* (Cohen *et al.*, 2004 ▶), which traced and built side chains for 161 residues (94% of the structure). Model adjustments and completion were performed with *Coot* (Emsley & Cowtan, 2004 ▶). Structure refinement was carried out using *REFMAC* v.5.5.0110 and included one TLS group and experimental phase restraints in the form of Hendrickson–Lattman coefficients from *SHARP* (Pannu *et al.*, 1998 ▶; Winn *et al.*, 2003 ▶). Data-reduction and refinement statistics for YP_001813558.1 are summarized in Table 1 ▶.

### 2.3. Validation and deposition

**Figure 1:**
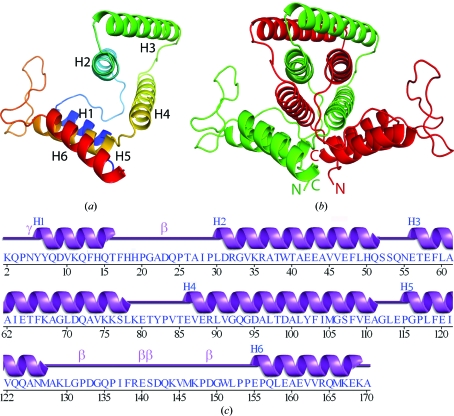
Crystal structure of YP_001813558.1 from *E. sibiricum* 255-15. (*a*) Ribbon diagram of the YP_001813558.1 protomer in the asymmetric unit, color-coded from the N-terminus (blue) to the C-terminus (red). Helices H1–H6 are indicated. (*b*) The novel dimeric assembly of YP_001813558.1 generated by helical segment swapping. Green and red tracings represent chain *A* and the symmetry-related chain *A*′ that form the dimer. The N- and C-termini are labeled. (*c*) Diagram showing the secondary-structure elements of YP_001813558.1 superimposed on its primary sequence. The α-helices (H1–H6), β-turns (β) and γ-turn (γ) are indicated.

The quality of the crystal structure was analyzed using the *JCSG Quality Control* server (http://smb.slac.stanford.edu/jcsg/QC). This server processes the coordinates and data through a variety of validation tools including *AutoDepInputTool* (Yang *et al.*, 2004 ▶), *MolProbity* (Chen *et al.*, 2010 ▶), *WHAT IF* v.5.0 (Vriend, 1990 ▶), *RESOLVE* (Terwilliger, 2003 ▶), *MOLEMAN*2 (Kleywegt, 2000 ▶) as well as several in-house scripts and summarizes the results. Protein quaternary structure analysis used the *PISA* server (Krissinel & Henrick, 2007 ▶). Fig. 1 ▶(*c*) was adapted from an analysis using *PDBsum* (Laskowski *et al.*, 2005 ▶); all others were prepared with *PyMOL* (DeLano Scientific). Atomic coordinates and experimental structure factors for YP_001813558.1 have been deposited in the PDB (PDB code 3nl9).

## 3. Results and discussion

### 3.1. Overall structure

The crystal structure of YP_001813558.1 was determined to 1.78 Å resolution using the MAD method (Fig. 1 ▶ *a*). Data-collection, model and refinement statistics for the YP_001813558.1 structure are summarized in Table 1 ▶. The asymmetric unit contains one YP_001813558.1 molecule (residues 2–170), *i.e.* one half of the linked crystallographic dimer (Fig. 1*b* ▶), two 1,2-ethanediol molecules and 141 water molecules. Residues Gly0 (remaining after cleavage of the expression and purification tag) and SeMet1 and side-chain atoms of Lys2, Gln72, Lys76, Lys79, Glu140 and Ser141 had poorly defined or no electron density and were omitted from the model. The Matthews coefficient (*V*_M_) for YP_001813558.1 was 2.2 Å^3^ Da^−1^, with an estimated solvent content of 44.0% (Matthews, 1968 ▶). The Ramachandran plot produced by *MolProbity* (Chen *et al.*, 2010 ▶) indicated that 98.8% of the residues are in favored regions, with no outliers. YP_001813558.1 is an all-α structure containing six α-helices (H1–H6, Fig. 1 ▶ *a*), with a total α-helical content of 64.5%.

**Figure 2:**
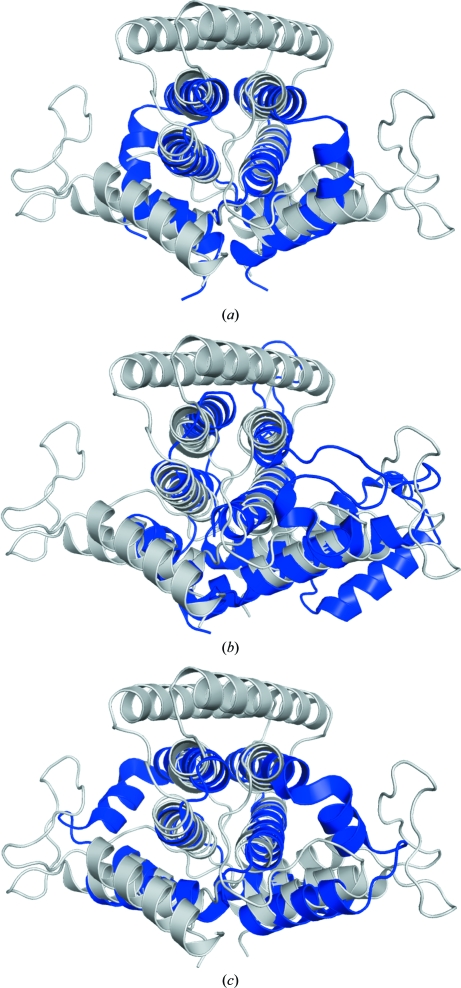
Superposition of the YP_001813558.1 biological dimer (gray) with other α-helical NTPases (blue): (*a*) *S. solfataricus* MazG (PDB code 1vmg; biological dimer), (*b*) *C. jejuni* dUTPase (PDB code 1w2y; single protomer, *i.e.* half of the biological dimer), (*c*) *B. cereus* PRATP-PH (PDB code 1yvw; dimer, *i.e.* half of the biological tetramer).

*PSI-BLAST* (Altschul *et al.*, 1997 ▶) and *FFAS* (Jaroszewski *et al.*, 2000 ▶) searches both detected similarities between YP_001813558.1 and other NTPases, such as the PRATP-PH family NTPases [PDB codes 1yvw (J. Benach, A. P. Kuzin, F. Forouhar, M. Abashidze, S. M. Vorobiev, R. Shastry, X. Rong, T. B. Acton, G. T. Montelione & J. F. Hunt, unpublished work), 2a7w (J. Benach, F. Forouhar, A. P. Kuzin, M. Abashidze, S. M. Vorobiev, X. Rong, T. B. Acton, G. T. Montelione & J. F. Hunt, unpublished work), 1y6x (Javid-Majd *et al.*, 2008 ▶), 1yxb (J. Benach, A. P. Kuzin, F. Forouhar, M. Abashidze, S. M. Vorobiev, X. Rong, T. B. Acton, G. T. Montelione & J. F. Hunt, unpublished work)], MazG NTPases [PDB codes 1vmg (Joint Center for Structural Genomics, unpublished work), 2a3q (Center for Eukaryotic Structural Genomics, unpublished work), 2q5z and 2q73 (Robinson *et al.*, 2007 ▶)], *Bacillus subtilis* NTPase YPJD (PDB code 2gta; S. M. Vorobiev, W. Zhou, J. Seetharaman, D. Wang, L. C. Ma, T. Acton, R. Xio, G. T. Montelione, L. Tong & J. F. Hunt, unpublished work) and the RS21-C6 core segment RSCUT, which has been reported to have NTPase activity (PDB code 2oie; Wu *et al.*, 2007 ▶). Superimposition of these structures onto the YP_001813558.1 crystallographic dimer shows that the general topology of the four-helix bundle is conserved; for example, the equivalent secondary elements of PRATP-PH from *B. cereus* (PDB code 1yvw) can be aligned with an r.m.s.d. of 2.5 Å (for 81 of 90 C^α^ atoms). Similarly, MazG from *Sulfolobus solfataricus* (PDB code 1vmg) can be superimposed onto YP_001813558.1 with an r.m.s.d. of 2.3 Å for 77 of 80 C^α^ atoms. MazG from *E. coli* can hydrolyze all eight of the canonical ribonucleoside and deoxy­nucleoside triphosphates to their respective monophosphates and PP_i_, with a preference for deoxynucleotides (Zhang & Inouye, 2002 ▶). YP_001813558.1 (170 residues) is significantly larger than MazG (PDB code 1vmg; 83 residues) and PRATP-PH (PDB code 1yvw; 95 residues) primarily owing to the presence of two additional helices, H3 located at the top of the four-helix bundle and H6 located at the C-terminus, and a long mostly unstructured loop between H1 and H2 (residues 17–29) that is α-helical and significantly shorter in both MazG (PDB code 1vmg; residues 23–33) and PRATP-PH (PDB code 1yvw; residues 23–32) (see Figs. 2 ▶ *a* and 2 ▶ *c*).

An initial *DALI* (Holm *et al.*, 2008 ▶) search for homologues of YP_001813558.1 did not identify any significant matches owing to the unusual segment swapping; however, a search with the MazG dimer (PDB code 1vmg) revealed structural similarities to the dUTPases 2cic (*Z* score 10.4; r.m.s.d. 3.3 Å; 139 C^α^ atoms aligned; O. V. Moroz, M. J. Fogg, D. Gonzalez-Pacanowska & K. S. Wilson, unpublished work), 1w2y (*Z* score 10.1, r.m.s.d. 3.4 Å, 139 C^α^ atoms aligned; Moroz *et al.*, 2004 ▶) and 2cje (*Z* score 8.2; r.m.s.d. 2.9 Å; 121 C^α^ atoms aligned; O. V. Moroz, M. J. Fogg, D. Gonzalez-Pacanowska & K. S. Wilson, unpublished work) and, of course, to other MazG NTP proteins, 2q73 (*Z* score 8.2; r.m.s.d. 1.5 Å; 77 C^α^ atoms aligned; Robinson *et al.*, 2007 ▶) and 2q5z (*Z* score 7.8, r.m.s.d. 1.7 Å, 78 C^α^ atoms aligned; Robinson *et al.*, 2007 ▶). Comparison of the superimposed YP_001813558.1 and *C. jejuni* dUTPase (PDB code 1w2y) structures shows that the H3 helix of YP_001813558.1 is absent in the 1w2y structure and the loops between helices in the two structures are very different. In addition, 1w2y contains an additional helix at the C-­terminus (Fig. 2 ▶ *b*) that is not found in YP_001813558.1.

### 3.2. Linked dimer

**Figure 3:**
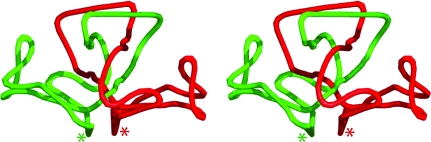
Simplified traces of the YP_001813558.1 linked dimer. Stereoview of the crystallographic dimer with the same orientation and color scheme as in Fig. 1 ▶(*b*) showing the inter-linked dimer. Note that in this representation the N- and C-termini of each monomer are joined in order to highlight the linked dimer. The linked N- and C-termini are marked with an asterisk. Smoothed curves were calculated as described previously (Norcross & Yeates, 2006 ▶).

The crystallographic structure of YP_001813558.1 displays a very unusual interlaced segment-swapped dimer, which implies that this obligatory dimer assembly is important for its function (Fig. 3 ▶). Size-exclusion chromatography combined with static light scattering confirmed that the dimer is the major oligomeric state in solution. Initial concerns that the segment-swapped dimer may have arisen from incorrect tracing of the model were eliminated by independent tracing of a SAD data set collected from a different crystal, which also resulted in a segment-swapped dimer. Interestingly, this intertwined dimer does not result in a knotted protein. In other words, the polypeptide chain would not form a knot if the C-terminus of chain *A* were joined to the N-terminus of chain *B* and the N- and C-termini of the resulting structure were pulled apart. This is notable because some knotted proteins are believed to have evolved by gene duplication and fusion of intertwined dimers (Bolinger *et al.*, 2010 ▶). In the present case, such a duplication would not lead to a knotted structure, despite the highly intertwined nature of the chains.

**Figure 4:**
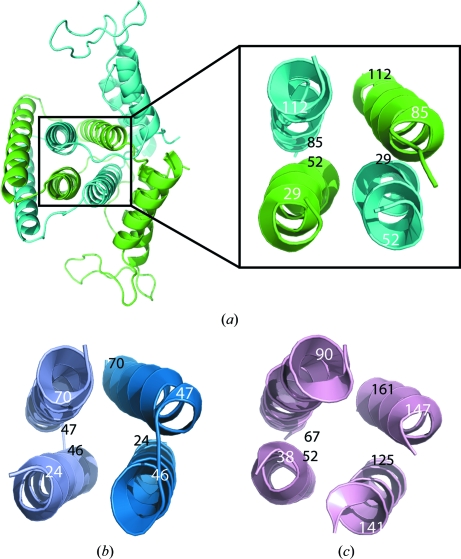
Comparison of the core four-helix bundles from the α-helical NTPase superfamily. These four-helix bundles either assemble upon dimerization or are present in a single monomer, resulting in the same down–up–down–up topology. White numbers are closest to the viewer and black numbers are farthest away. (*a*) Ribbon diagram showing the dimer of YP_001813558.1. (*b*) Ribbon diagram showing the central four helices of *S. solfataricus* MazG. (*c*) Ribbon diagram showing the central four helices from a single protomer of *C. jejuni* dUTPase.

A surface area of 5104 Å^2^ per monomer is buried upon dimer formation. The conserved central four helices that form part of the active site are helices H2 (residues 30–52) and H4 (residues 86–111) from chain *A* and the equivalent helices from its symmetry-related partner (chain *A*′) and are assembled in a down–up–down–up topology (Fig. 4 ▶ *a*). The core of the *S. solfataricus* MazG (PDB code 1vmg) structure also consists of a dimeric four-helix bundle with each monomer contributing two helices (Fig. 4 ▶ *b*), but in a different arrangement that appears to represent a minimal functional unit for dUTPases (Moroz *et al.*, 2004 ▶). The four-helix bundle of the *C. jejuni* dUTPase (PDB code 1w2y) is contained within a single protomer (Fig. 4 ▶ *c*); thus, dUTPases are thought to have evolved from MazG-like ancestors by gene duplication (Moroz *et al.*, 2005 ▶). The central core four-helix bundle from PRATP-PH also reveals a similar down–up–down–up topology, as shown in Fig. 4 ▶(*c*).

### 3.3. Putative metal-binding site predicted from the homolog structures

**Figure 5:**
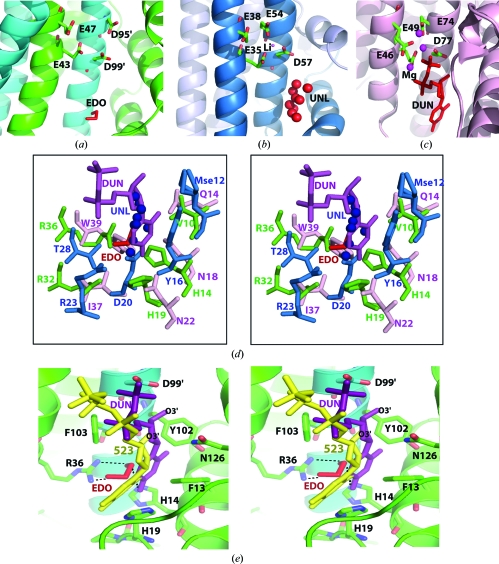
(*a*–*c*) Comparison of the active sites of YP_001813558.1, *S. solfataricus* MazG and *C. jejuni* dUTPase. The putative conserved active-site metal-binding residues are shown as stick models. Note that Asp95 in YP_001813558.1 is offset by one residue when compared with the other two structures. No metal was found in YP_001813558.1. One Li^+^ ion (red ball) is bound in MazG based on the crystallization conditions. Three Mg^2+^ ions (red balls) are bound in the *C. jejuni* dUTPase structure. The nucleotide-binding sites contain either a 1,2-ethanediol (EDO) molecule (YP_001813558.1), an unknown ligand (UNL; *S. solfataricus* MazG) or 2′-deoxyuridine 5′-α,β-imidodiphosphate (DUN; dUTPase; PDB code 1w2y) and are represented in red. (*d*) Comparison of the nucleotide-recognition site in YP_001813558.1 (green), *S. solfataricus* MazG (light blue) and *C. jejuni* dUTPase (pink) as a stereoview. The EDO molecule from YP_001813558.1 (red sticks), UNL from *S. solfataricus* MazG (blue balls) and DUN from *C. jejuni* dUTPase (purple sticks) are shown. Mse12 is modeled as three conformations in the MazG structure. (*e*) Stereoview of the superposition of the substrate analogs DUN (purple) from *C. jejuni* dUTPase and 2-deoxy-5-methylcytidine-5′-(tetrahydrogen triphosphate (yellow) from *M. musculus* RS21-C6 and the EDO (red) molecule bound to the YP_001813558.1 structure. Hydrogen bonds are shown as dotted lines. The key residues from YP_001813558.1 that are predicted to be involved in substrate binding are presented as a green stick model.

The location of the potential metal-binding site in YP_001813558.1 and MazG was deduced based on homology with the structure of *C. jejuni* dUTPase with a substrate analog bound to the active site. Divalent cations, preferably magnesium, are essential for NTPase activity (Nyman, 2001 ▶). Interestingly, although the YP_001813558.1 active site assembles quite differently from those of the other NTPases, the putative metal-binding sites in all three proteins are absolutely conserved, except for a one-residue offset of Asp95 in YP_001813558.1. This potential metal-binding site is formed by Glu43 and Glu47 in H2 of chain *A* and by Asp95 and Asp99 in H4 of the symmetry-related chain in the dimer (Fig. 5 ▶ *a*). A symmetry-related site is obviously formed on the opposite side of the dimer from the twofold operation. The metal-binding residues in *S. solfataricus* MazG (PDB code 1vmg) are Glu35, Glu38, Glu54 and Asp57 (Fig. 5 ▶ *b*). In dUTPase (PDB code 1w2y), which is related to MazG (PDB code 1vmg) by an ancestral duplication, the metal-binding residues are Glu46, Glu49, Glu74 and Asp77 (Fig. 5 ▶ *c*). The metal-binding residues, 2′-deoxyuridine 5′-α,β-imidodiphosphate (DUN) and waters participate in the octahedral coordination of Mg ions with distances that range from 1.86 to 2.25 Å.

### 3.4. Nucleotide-binding site

In *C. jejuni* dUTPase, Asp77 plays a central role in substrate binding. In addition to coordinating the Mg^2+^ ion and binding the terminal phosphate of the substrate analog 2′-deoxyuridine 5′-α,β-imidodiphosphate (DUN), Asp77 also binds the ribosyl 3′-OH group (Moroz *et al.*, 2004 ▶). In *Mus musculus* RS21-C6, the binding mode of the terminal phosphate is significantly different compared with that of *C. jejuni* dUTPase, presumably owing to the absence of magnesium ions. However, Asp98 (equivalent to Asp77) is located close to the bound 2-deoxy-5-methylcytidine-5′-(tetrahydrogen triphosphate) and binds to the ribosyl 3′-OH group of the nucleoside moiety *via* a water-mediated interaction (Wu *et al.*, 2007 ▶). Therefore, it is thought that the corresponding conserved residues, Asp99 in YP_001813558.1 and Asp57 in *S. solfataricus* MazG, perform similar roles in these enzymes. Another important residue for recognition of the substrate ribosyl 3′-OH in *C. jejuni* dUTPase is Asn179. This residue is conserved in both YP_001813558.1 (Asn126) and *M. musculus* RS21-C6 (Asn125), but not in *S. solfataricus* MazG.

In YP_001813558.1, the putative sugar-binding residues are Tyr102 and Phe103, between which the sugar moiety is sandwiched, and Asn126, which discriminates between deoxyribose and ribose (Fig. 5 ▶ *e*). The latter is conserved in most members of the all-α-helical NTP pyrophosphatase superfamily that have been shown to have a preference for dNTP (the dUTPase, dCTPase and RS21-C6 families), but is not conserved in the ribonucleosidetriphosphate-hydrolyzing HisE and EcMazG families (Nonaka *et al.*, 2009 ▶; Robinson *et al.*, 2007 ▶). Neither the YP_001813558.1 nor the *S. solfataricus* MazG structures have known biological ligands in their nucleotide binding sites (Fig. 5 ▶ *d*). The YP_001813558.1 structure contains a 1,2-ethanediol molecule and the *S. solfataricus* MazG structures contain an unidentified ligand (UNL) in the nucleotide-binding site. Since those ligands could mimic nucleotide substrates (Fig. 5 ▶ *d*), we speculate that both YP_001813558.1 and *S. solfataricus* MazG enzymes can function as dNTPases.

The uracil-recognition site of *C. jejuni* dUTPase is formed by Gln14 N^∊2^, Asn18 O^δ1^ and Asn22 N^δ2^ and is not conserved in YP_001813558.1 or *S. solfataricus* MazG. The corresponding residues in YP_001813558.1 are Val10, His14 and His19; His14 N^∊2^ is hydrogen bonded to the O2 atom of a 1,2-ethanediol molecule in the ligand-binding site. The corresponding region in the *S. solfataricus* MazG structure contains Mse12, which adopts three side-chain conformations, Tyr16 and Asp20, where Asp20 O^δ1^ and Asp20 O^δ2^ interact with the O7 and O9 atoms of the UNL ligand, respectively. Thus, it appears that YP_001813558.1 and *S. solfataricus* MazG may not bind uracil (Fig. 5 ▶ *d*). The major determinant of the substrate specificity involved in base recognition in YP_001813558.1 would be Arg36, where Arg36 N^η1^ and Arg36 N^η2^ interact with the O1 and O2 atoms of the 1,2-ethanediol molecule, respectively (Fig. 5 ▶ *e*). Arg36 provides two hydrogen-bond donors that could interact with two adjacent acceptors on the base. Of the canonical bases, only cytosine would satisfy these conditions for making two hydrogen bonds. Therefore, potential substrates for YP_001813558.1 include dCTP and its derivatives (*e.g.* 5-methyl or 5-hydroxymethyl dCTP). In addition, the two modified bases *O* ^4^-methylthymine and 8-hydroxy­guanine are also predicted to interact in the same manner as cytosine. These modified bases could provide additional hydrogen bonds from O4 of *O* ^4^-methylthymine (or O6 of 8-hydroxyguanine) to His19 and/or Arg32 of YP_001813558.1.

The pyrophosphate-recognition residues of *C. jejuni* dUTPase are mostly conserved in YP_001813558.1 and *S. solfataricus* MazG, except for the C-terminal region. Lys175 of *C. jejuni* dUTPase is structurally equivalent to Val122 of YP_001813558.1 and Lys80 of MazG. This residue is located in the loop region near the C-terminus of *C. jejuni* dUTPase, which also contains the pyrophosphate-recognition residues Arg182, Tyr187, Lys194 and Asn202. This region does not superimpose well in YP_001813558.1 and is absent in MazG. The corresponding pyrophosphate-recognition loop in YP_001813558.1 is located between H5 and H6. This loop and the two neighboring C-­terminal helices (H5 and H6) of YP_001813558.1 are in an open conformation and are more exposed to solvent compared with the equivalent region in *C. jejuni* dUTPase, which may suggest an induced-fit mechanism for substrate binding involving movement of the C-terminal region.

## 4. Conclusions

We report a very unusual segment-swapped linked-dimer structure of a dUTPase from *E. sibiricum* 255-15, which implies that this obligatory dimer assembly is important for its function of adaptation to an extreme cold environment. Unusual, covalently interlinked dimeric structures have been implicated previously in stabilizing proteins (Boutz *et al.*, 2007 ▶; Duff *et al.*, 2003 ▶). Structural analysis and comparisons indicate that YP_001813558.1 is a dNTPase that potentially prefers dCTPs or its derivatives. Further biochemical analyses are needed to confirm these predictions. The availability of further sequences and structures of NTP pyrophosphohydrolases should shed light on the evolutionary history of this intriguing protein family. The information presented here, in combination with further biochemical and biophysical studies, should yield valuable insights into the functional role of YP_001813558.1. Additional information about YP_001813558.1 is available from TOPSAN (Krishna *et al.*, 2010 ▶) at http://www.topsan.org/explore?PDBid=3nl9.

## Supplementary Material

PDB reference: putative NTP pyrophospho­hydrolase, 3nl9

## Figures and Tables

**Table 1:** Summary of crystal parameters, data-collection and refinement statistics for YP_001813558.1 (PDB code 3nl9) Values in parentheses are for the highest resolution shell.

	λ_1_ MADSe	λ_2_ MADSe
Crystal parameters		
Space group	*C*2	
Unit-cell parameters (Å, °)	*a* = 52.09, *b* = 69.04, *c* = 50.21, β = 111.8	
Mosaicity (°)	0.91	
Data collection		
Wavelength (Å)	1.0000	0.9798
Resolution range (Å)	39.6–1.78 (1.83–1.78)	39.6–1.78 (1.83–1.78)
No. of observations	43073	43110
No. of unique reflections	15531	15528
Completeness (%)	98.1 (97.9)	98.1 (97.3)
Mean *I*/σ(*I*)	9.8 (2.1)	8.6 (1.8)
*R*_merge_ on *I*†	0.069 (0.555)	0.082 (0.563)
*R*_meas_ on *I*‡	0.086 (0.687)	0.102 (0.698)
*R*_p.i.m._ on *I*§	0.050 (0.401)	0.059 (0.408)
Overall *B* factor from Wilson plot (Å^2^)	21.3	21.0
Model and refinement statistics		
Data set used in refinement	λ_1_ MADSe	
Resolution range (Å)	39.6–1.78	
No. of reflections (total)	15531	
No. of reflections (test)	788	
Completeness (%)	97.8	
Cutoff criterion	|*F*| > 0	
*R*_cryst_¶	0.177	
*R*_free_††	0.222	
Stereochemical parameters		
Restraints (r.m.s.d. observed)		
Bond angles (°)	1.30	
Bond lengths (Å)	0.015	
Average protein isotropic *B* value (Å^2^)	26.0‡‡	
Average solvent isotropic *B* value (Å^2^)	33.6	
ESU§§ based on *R*_free_ (Å)	0.14	
Protein residues/atoms	169/1340	
Water/cryoprotectant molecules	141/2	

† *R*_merge_ = 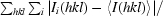 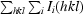.

‡ The redundancy-independent (multiplicity-weighted) merging *R* factor, *R*_meas_ = 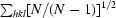 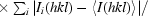 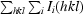 (Diederichs & Karplus, 1997 ▶).

§ The precision-indicating merging *R* factor, *R*_p.i.m._ = 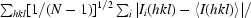 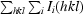 (Weiss & Hilgenfeld, 1997 ▶; Weiss *et al.*, 1998 ▶).

¶ *R*_cryst_ = 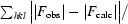 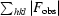, where *F*_calc_ and *F*_obs_ are the calculated and observed structure-factor amplitudes, respectively,

†† *R*_free_ is the same as *R*_cryst_ but for 5.1% of the total reflections chosen at random and omitted from refinement

‡‡ This value represents the total *B* and includes both TLS and residual *B* components.

§§ Estimated overall coordinate error (Collaborative Computational Project, Number 4, 1994 ▶; Cruickshank, 1999 ▶).
